# Early Discrimination and Prediction of *C. fimbriata*-Infected Sweetpotatoes during the Asymptomatic Period Using Electronic Nose

**DOI:** 10.3390/foods11131919

**Published:** 2022-06-28

**Authors:** Jiawen Wu, Linjiang Pang, Xiaoqiong Zhang, Xinghua Lu, Liqing Yin, Guoquan Lu, Jiyu Cheng

**Affiliations:** College of Food and Health, Zhejiang A&F University, Hangzhou 311300, China; wjw_1006@163.com (J.W.); xqzhang@stu.zafu.edu.cn (X.Z.); xhlu@zafu.edu.cn (X.L.); 20220027@zafu.edu.cn (L.Y.); lugq10@zju.edu.cn (G.L.); jy_ch@163.com (J.C.)

**Keywords:** electronic nose, sweetpotato, infection, volatile compounds, asymptomatic period, rapid detection

## Abstract

Sweetpotato is prone to disease caused by *C. fimbriata* without obvious lesions on the surface in the early period of infection. Therefore, it is necessary to explore the possibility of developing an efficient early disease detection method for sweetpotatoes that can be used before symptoms are observed. In this study, sweetpotatoes were inoculated with *C. fimbriata* and stored for different lengths of time. The total colony count was detected every 8 h; HS-SPME/GC–MS and E-nose were used simultaneously to detect volatile compounds. The results indicated that the growth of *C. fimbriata* entered the exponential phase at 48 h, resulting in significant differences in concentrations of volatile compounds in infected sweetpotatoes at different times, especially toxic ipomeamarone in ketones. The contents of volatile compounds were related to the responses of the sensors. E-nose was combined with multiple chemometrics methods to discriminate and predict infected sweetpotatoes at 0 h, 48 h, 64 h, and 72 h. Among the methods used, linear discriminant analysis (LDA) had the best discriminant effect, with sensitivity, specificity, precision, and accuracy scores of 100%. E-nose combined with K-nearest neighbours (KNN) achieved the best predictions for ipomeamarone contents and total colony counts. This study illustrates that E-nose is a feasible and promising technology for the early detection of *C. fimbriata* infection in sweetpotatoes during the asymptomatic period.

## 1. Introduction

Sweetpotato (*Ipomoea batatas* L. Lam) is the sixth largest crop in the world and is rich in carbohydrates, dietary fibre, vitamins, minerals, and bioactive compounds (such as phenolic acids, flavonoids, and carotenoids) [1,2]. Sweetpotatoes are grown in more than 100 countries worldwide, mainly in Asia and Africa. China has the largest sweetpotato plantation areas in the world [3,4]. According to FAO statistics, in 2020, sweetpotato planting areas in China covered 2.25 million square kilometres, and the total output was about 49 million tons, representing 30.40% and 54.97% of the world sweetpotato planting area and output, respectively. Compared with other crops, sweetpotatoes can support more people per unit of land, and they are the staple food for large populations over the world [5]. However, sweetpotatoes are prone to diseases during postharvest storage and transportation, such as black rot [6], soft rot [7], and root rot [8]. Among them, black rot is the most typical. It is estimated that about 10% to 20% of sweetpotatoes are lost due to black rot disease during postharvest storage and transportation each year [9]. Black rot is caused by *Ceratocystis fimbriata* Ellis & Halsted (*C. fimbriata*) infection [10], which usually occurs on the wounds and lenticels of sweetpotatoes [11]. The initial symptom is the presence of black, round, slightly depressed spots. With the advancement of infection, the black spots gradually expand, the depressions increase, and black bristles appear [12]. The disease eventually leads to wilting, ulcers, and rot [13]. After sweetpotatoes are infected with *C. fimbriata*, large numbers of toxins are produced during the disease process [14], mainly ipomeamarone [15], which is seriously harmful to human health. The LD_50_ in mice is 230 mg/kg body weight [16]. Ipomeamarone can cause liver and lung poisoning in humans and animals [17,18], resulting in respiratory distress, pulmonary oedema, congestion, and even livestock death [19].

At present, the discrimination of postharvest disease of sweetpotatoes relies mainly on manual work. However, there are no obvious symptoms on the surface of *C. fimbriata*-infected sweetpotatoes in the early period, resulting in inaccurate identification of infected products as acceptable. Only by removing the infected sweetpotatoes during the incubation period of the disease can large-scale rot be reduced to the greatest extent possible during storage and the economic loss mitigated. It is of great significance to explore the development of a fast, accurate, and non-destructive method of sweetpotato disease detection for use before symptoms are observed on the surface.

When agricultural products are infected by pathogens, normal physiological metabolism is disturbed, causing volatile compound contents to change significantly [20,21,22]. Therefore, the disease condition can be determined by detecting changes in volatile compounds in *C. fimbriata*-infected sweetpotatoes. At present, the main detection technologies for volatile compounds in agriculture include gas chromatography–mass spectrometry (GC–MS) [23], gas chromatography–ion mobility spectrometry (GC–IMS) [24], and gas chromatography–olfactometry (GC–O) [25]. These technologies have high accuracy; however, there are some disadvantages to these methods, including cumbersome operation, high costs, and long detection times [26]. Therefore, these technologies are usually combined with other rapid detection methods, such as electronic nose (E-nose) and hyperspectral imaging, to achieve rapid detection and accurate identification [27]. Simultaneously, quantitative prediction of physiological indicators can also be achieved [28].

As a non-destructive, rapid, sensitive, and real-time volatile compounds detection technology, E-nose has been widely used in postharvest quality control, freshness measurement, disease detection, and pest infestation of agricultural products [29,30,31]. For example, E-nose has achieved excellent results in detecting citrus fruit *Bactrocera dorsalis* infection [32], garlic *Alternariaembellisia* infection [33], and pine mushroom *Aspergillus niger* infection [34]. In the quantitation of toxins, E-nose has been used to quantify aflatoxin B_1_ and fumonisin in corn [35] and has been combined with Raman microspectroscopy to quantify pesticide residues in tea [36].

At present, most of the applications of E-nose are used to discriminate and monitor agricultural product disease when obvious lesion symptoms have appeared on the surfaces of crops. There has been no previously reported study on the use of E-nose for the detection of *C. fimbriata* infection in sweetpotatoes during the surface asymptomatic period (0 to 72 h). Therefore, in this study, E-nose was used to obtain volatile compounds and was combined with chemometrics to establish a rapid discriminant model of *C. fimbriata*-infected sweetpotatoes. The volatile compounds were also used to predict the content of sweetpotato toxins and the total colony count to evaluate the feasibility of E-nose technology for early warning of sweetpotato disease. This study provided a new method for real-time detection of *C. fimbriata* infection and toxin production in sweetpotatoes during the asymptomatic period.

## 2. Materials and Methods

### 2.1. Sample Preparation

The pathogenic fungi *C. fimbriata* was provided by the Xuzhou Academy of Agricultural Sciences in Xuzhou, China. In a sterile environment, *C. fimbriata* was inoculated on potato dextrose agar (PDA) and cultured at 28 °C and 85% relative humidity for 7 to 14 days. The spores on the surface of the medium were rinsed with sterile saline solution (0.85% NaCl; all solution concentrations are volume/volume). Then, the spore suspension was collected in a conical flask and counted with a haemocytometer under a microscope. The spore suspension was adjusted to a final concentration of 1 × 10^6^ spores mL^−1^ with sterile saline solution.

Sweetpotatoes of the variety “Xinxiang” were provided by the sweetpotato planting base of the crop research institute at Zhejiang A&F University in Hangzhou, China. Fresh sweetpotatoes of the same colour and size without diseases or insect pests were selected. The sweetpotatoes were first washed with tap water and soaked in 1% sodium hypochlorite solution for 2 min to disinfect them, then rinsed with purified water, and, finally, 75% ethanol solution was used to disinfect the surface of the sweetpotatoes and they were dried. Six holes were punched evenly in the equatorial part of the sweetpotatoes; each hole was 2 mm in diameter and 5 mm in depth. All holes were inoculated with 10 µL of spore suspension. After the spore suspension was absorbed, sweetpotato samples were placed in a sterilized incubator at 28 °C and 80% relative humidity and taken for detection at 0 h, 8 h, 16 h, 24 h, 32 h, 40 h, 48 h, 56 h, 64 h, and 72 h. Sweetpotatoes inoculated with sterile saline solution at 0 h were used as control.

### 2.2. Colony Count

The detection method of the total colony count followed the method of Zhang et al. [37], with appropriate modifications. The colonies were counted as colony-forming units (CFUs) and presented as CFU g^−1^.

### 2.3. Headspace Solid-Phase Microextraction/Gas Chromatography–Mass Spectrometry (HS-SPME/GC–MS) Detection

Volatile compounds in sweetpotatoes were analysed by HS-SPME/GC–MS technology. The method was referred to Lan et al. [38], with appropriate modifications. A DVB/CAR/PDMS (50/30 µm, Supelco, St. Louis, MO, USA) fibre was used to extract and concentrate the volatile compounds. Then, the volatile compounds were separated and identified by QP-2010 GC–MS (Shimadzu, Kyoto, Japan). Accurately weighed, 3.0 g of chopped sweetpotato tissue was used as the sample, and 5 µL of 2-octanol (0.1 µL mL^−1^, as an internal standard) was added to a 20 mL headspace vial, which was then sealed with a silicone rubber lid. After equilibration in a water bath at 70 °C for 15 min, the fibre was inserted into the headspace vial. The fibre tip was pushed out and exposed 1 cm above the samples at 70 °C for 45 min. Finally, the fibre was injected into the GC for desorption at 250 °C for 5 min and the data collection was started at the same time.

An HP-5 capillary column (30 m × 0.25 mm, 0.25 µm) was used in the GC. The injection port temperature was 250 °C. Helium was used as the carrier gas at a constant flow rate of 1.0 mL min^−1^. The splitless injection was carried out with the following heating procedure of the column. The initial temperature was set at 45 °C and held for 3 min. Then, the temperature was increased to 125 °C at a constant ramp-up rate of 5 °C min^−1^. Subsequently, the temperature was increased to 175 °C at a constant ramp-up rate of 3°C min^−1^ and held for 3 min. Finally, the temperature was increased to 230 °C at a constant ramp-up rate of 10 °C min^−1^ and held for 2 min. The MS connector temperature was 280 °C. The MS was operated in the electron impact ionization mode (70 eV), and the data were scanned from 50 to 550 *m*/*z*. The temperature of the ion source was 230 °C, and the solvent was extended for 2 min.

The volatile compounds were identified by comparison with National Institute of Standards and Technology (NIST) standards, and the substances with matching degrees greater than 80% were screened out [39]. The internal standard of 2-octanol was used to quantify the volatile compounds in sweetpotatoes. The data for each group were from three replicate experiments and the results were presented as the means ± standard deviation (SD) ng g^−1^ fresh weight (FW) equivalent of 2-octanol.

### 2.4. E-Nose Detection

**Figure 1:**
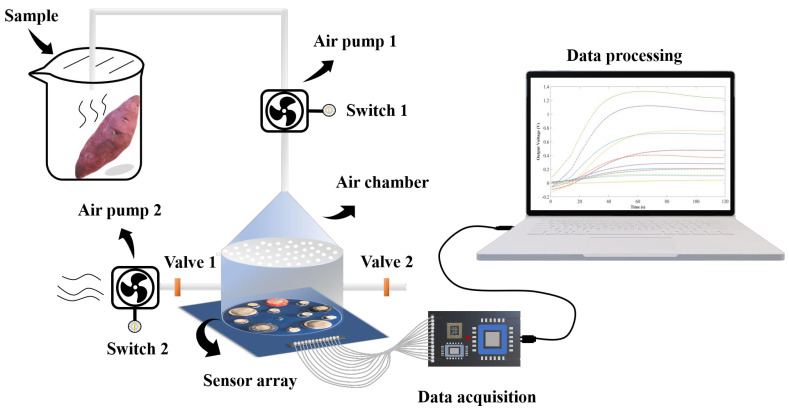
Composition and working principle of E-nose.

E-nose was self-assembled by the agricultural products storage laboratory of Zhejiang A&F University in Hangzhou, China. As shown in Figure 1, it consisted of an electromechanical control unit, a sensor array, and a data acquisition unit. The volume of the air chamber was 350 mL. The sensor array consisted of 12 metal oxide sensors and one temperature and humidity sensor to monitor environmental temperature and humidity. The metal oxide sensors were purchased from Figaro Engineering Corporation, Japan. The models of the sensor were TGS2620, TGS826, TGS822, TGS832, TGS813, TGS816, TGS2600, TGS2610, TGS2611, TGS2615, TGS2602, and TGS2603, numbered S1, S2, S3, S4, S5, S6, S7, S8, S9, S10, S11, and S12, respectively. The temperature and humidity sensor HTU20 was purchased from the Humirel company, France, and numbered S13. The sensitive gases of each sensor are shown in Table S1.

A dehumidifier was installed in the room to control the humidity and keep the ambient humidity constant. The relative humidity in the air chamber during sample testing is shown in Table S2. Detection with E-nose referred to the method of Pang et al. [40], with appropriate modifications. The prepared sweetpotato samples were put into 500 mL clean and odourless beakers, which were then sealed and placed in a constant-temperature environment of 30 °C for 15 min to obtain headspace gas. E-nose was preheated for 2 h in advance. When the sample was measured, the valves on both sides of the air chamber were closed, and air pump 1 was turned on. The headspace gas in the beaker was pumped out to the air chamber at a constant flow rate of 300 mL min^−1^ for 90 s, then equilibrated for 30 s. After the sample was detected, sweetpotato and beaker were removed. The valves on both sides of the air chamber were opened, air pumps 1 and 2 were turned on at the same time, and the airflow rate was adjusted to the maximum. Fresh air filtered with activated carbon was pumped into the air chamber for 120 s for cleaning. When the response curve returned to the baseline, the experiment proceeded to the next sample detection. Thirty sweetpotato samples were detected at each time point.

### 2.5. Data Analysis

IBM SPSS Statistics 22 and Origin 2021b were used to analyse the HS-SPME/GC–MS data, and Origin 2021b and MATLAB R2018b were used to analyse E-nose data.

To reduce the influence of ambient temperature and humidity, the signal was denoised and smoothed, and E-nose data were divided into calibration sets and prediction sets after removing outliers. The maximum response value [41] was selected as the extraction feature representing the processed signal for subsequent analysis. Principal component analysis (PCA), partial least squares discriminant analysis (PLS-DA), linear discriminant analysis (LDA), and classification and regression trees (CART) were used to establish early discrimination models of *C. fimbriata*-infected sweetpotatoes during the asymptomatic period. The discrimination performance of each model was evaluated by sensitivity (Sens), specificity (Spec), precision (Prec), and accuracy (Ac).

K-nearest neighbours (KNN), partial least squares (PLS), and principal component regression (PCR) were used to predict the content of ipomeamarone and total colony count for *C. fimbriata*-infected sweetpotatoes during the asymptomatic period. The prediction performance of each model was evaluated by correlation coefficient of determination ($R^{2}$) of calibration ($R_{c}{}^{2}$) and prediction ($R_{p}{}^{2}$), root mean square error (RMSE) of calibration (RMSEC) and prediction (RMSEP), and residual prediction deviation (RPD). Parameters were defined as follows:

$$\mathrm{Sens}=\frac{n_{\mathrm{gg}}}{n_{g}}$$

$$\mathrm{Spec}=\frac{\sum_{k=1}^{G}\left({n^{\prime }}_{k}-n_{\mathrm{gk}}\right)}{n-n_{g}}\;k\neq g\;\;\;\;\;\;{n^{\prime }}_{k}=\sum_{g=1}^{G}n_{\mathrm{gk}}$$

$$\mathrm{Prec}=\frac{n_{\mathrm{gg}}}{{n^{\prime }}_{g}}$$

$$\mathrm{AC}=\frac{\sum_{g-1}^{G}n_{\mathrm{gg}}}{n}$$

$$R_{c}{}^{2},R_{p}{}^{2}=1-\frac{\sum_{i=1}^{n}{\left(y_{i}-\hat{y_{i}}\right)}^{2}}{\sum_{i=1}^{n}{\left(y_{i}-\bar{y}\right)}^{2}}$$

$$\mathrm{RMSEC},\mathrm{RMSEP}=\sqrt{\frac{1}{n}\overset{n}{\underset{i=1}{\sum }}{\left(y_{i}-\hat{y_{i}}\right)}^{2}}$$

$$\mathrm{RPD}=\frac{\sqrt{\frac{1}{n-1}\sum_{i=1}^{n}{\left(y_{i}-\bar{y}\right)}^{2}}}{\sqrt{\frac{1}{n}\sum_{i=1}^{n}{\left(y_{i}-\hat{y_{i}}\right)}^{2}}}$$

where $n_{g}$ is the total number of samples belonging to the g-th class, $n_{\mathrm{gg}}$ is the number of samples belonging to class g and correctly assigned to class g, ${n^{\prime }}_{k}$ is the total number of samples assigned to the k-th class, ${n^{\prime }}_{g}$ is the total number of samples assigned to the g-th class, n is the total number of samples, $y_{i}$ and $\hat{y_{i}}$ are the measured and predicted values of the i-th sample, and $\bar{y}$ is the mean value of all samples in the calibration or prediction set.

## 3. Results and Discussion

### 3.1. Growth of C. fimbriata during Infection in Sweetpotatoes

**Figure 2:**
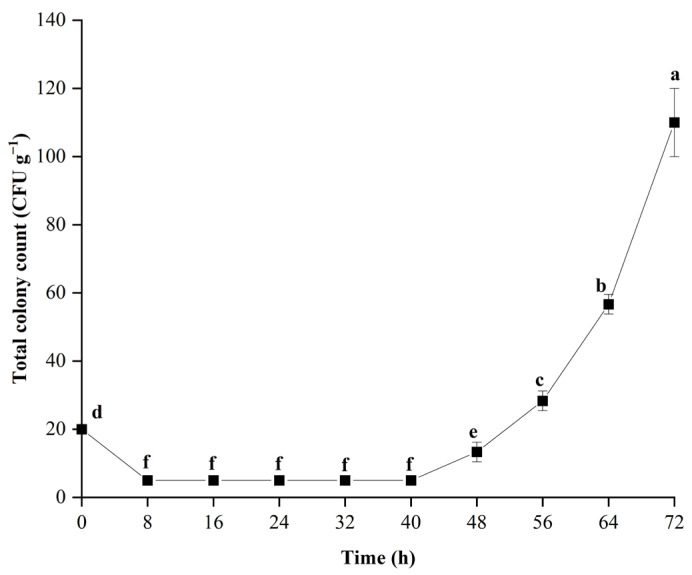
Fitting curve of *C. fimbriata* growth, showing the count of colony-forming units over time. The bars represent the standard deviations (±SD) of three independent measurements. Different lowercase letters represent significant differences according to Duncan’s multiple range test (*p* < 0.05).

The number of pathogens is an important factor affecting disease in sweetpotatoes. The growth curve of *C. fimbriata* in sweetpotatoes is shown in Figure 2. From 0 to 72 h, the total colony count showed a stable trend at first and then increased. Fungi growth can be divided into three phases: the lag phase, the exponential phase, and the stationary phase [42]. *C. fimbriata* exhibited a long lag phase (0 to 40 h) and entered the exponential phase from 48 h. The longer the infection time, the faster the growth rate. The total colony count was in a low stable state from 8 to 40 h, which indicated that the *C. fimbriata* needed to re-adapt to the new environment in the early period of inoculation [43] and begin synthesising enzymes, coenzymes, or intermediate metabolites necessary for growth. During that time, the total colony count did not increase significantly (*p* > 0.05). From 48 to 72 h, the spores of *C. fimbriata* began to proliferate, resulting in a rapid and significant increase in total colony count.

**Figure 3:**
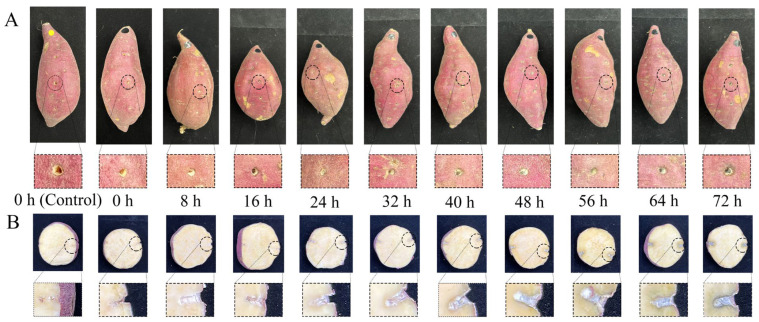
Pathological disease process of sweetpotatoes inoculated with *C. fimbriata* from 0 to 72 h. (**A**) Surface disease symptoms. (**B**) Internal disease symptoms.

It can be seen from Figure 3 that, from 0 to 40 h after inoculation with *C. fimbriata*, the surface and internal tissue of sweetpotatoes did not show obvious disease symptoms. However, from 48 h of infection, the internal tissue of sweetpotatoes showed slight black lesions. The disease became more serious with increasing infection time. After 72 h of infection, the surface of sweetpotatoes began to show symptoms of black spots, indicating that the infection of *C. fimbriata* in sweetpotatoes had spread from the internal tissue to the surface. When the lesion symptoms can be observed on the surface of sweetpotatoes, the internal tissue has become severely diseased. Therefore, it is of great significance to explore a technology for early identification of sweetpotato disease during storage.

### 3.2. Analysis of Volatile Compounds by HS-SPME/GC–MS

**Figure 4:**
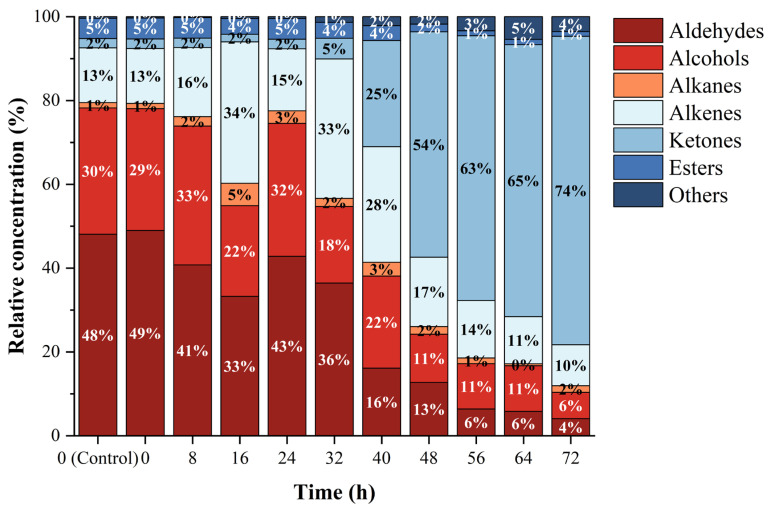
Relative concentrations of different categories of volatile compounds in the *C. fimbriata*-infected group and the control group from 0 to 72 h.

When agricultural products are infected by pathogens, they usually release volatile compounds that are obviously different from those that occur in a healthy state [44,45]. Using HS-SPME/GC–MS technology and an online search of NIST, a total of 65 kinds of volatile compounds were identified and quantified, as shown in Table S3, including 11 types of aldehydes, 11 types of alcohols, 7 types of alkanes, 19 types of alkenes, 7 types of ketones, 7 types of esters, and 3 types of other compounds. The changes in the contents of different categories of volatile compounds at 10 time points are shown in Table 1 and Figure 4. Results showed that there was no statistical difference in volatile compounds at 0 h between the *C. fimbriata*-infected group and the control group (*p* > 0.05). At the time of inoculation (0 h), the volatile compounds were mainly aldehydes, alcohols, and alkenes. The contents of these three categories of volatile compounds were significantly higher than those of alkanes, ketones, esters, and others (*p* < 0.05). From 48 to 72 h, the content of all categories of volatile compounds increased significantly, especially ketones. At 0 h, the content of ketones was extremely low—23.39 ng g^−1^ FW, accounting for only 2%. At 48 h, the content of ketones was 2932.65 ng g^−1^ FW, and at 72 h of infection, the content of ketones was as high as 17,346.42 ng g^−1^ FW, accounting for 74% of the total volatile compounds. This was followed by alkenes, with a content of 133.39 ng g^−1^ FW at 0 h, accounting for 13%. At 72 h, the content of alkenes was 2304.64 ng g^−1^ FW, accounting for 10%. The content showed a significant increase overall (*p* < 0.05) but fluctuated. At the time of inoculation (0 h), the content of alcohols was 296.54 ng g^−1^ FW, accounting for 29%. The content was relatively stable from 0 to 40 h, with no significant change (*p* > 0.05). At 48 h, the content began to increase significantly (*p* < 0.05), and at 72 h it was 1473.76 ng g^−1^ FW but the proportion had decreased to 6%. The content of aldehydes was 499.00 ng g^−1^ FW at 0 h and the ratio reached 49%. After infection with *C. fimbriata*, the aldehydes content showed a fluctuating upward trend. At the late period of infection (72 h), the content increased significantly (*p* < 0.05), reaching 963.91 ng g^−1^ FW, but the proportion had decreased to 4%. The alkanes and esters showed a significant upward trend from 0 to 72 h in general, but the proportion was always low. The content at 0 h was 12.59 ng g^−1^ FW and 50.95 ng g^−1^ FW, respectively, accounting for 1% and 5%. The content at 72 h after infection was 376.55 ng g^−1^ FW and 270.25 ng g^−1^ FW, accounting for 2% and 1%, respectively. In general, *C. fimbriata* infection led to the decrease or disappearance and increase or appearance of different types of volatile compounds, which was beneficial for the early discrimination of *C. fimbriata*-infected sweetpotatoes during the asymptomatic period.

**Figure 5:**
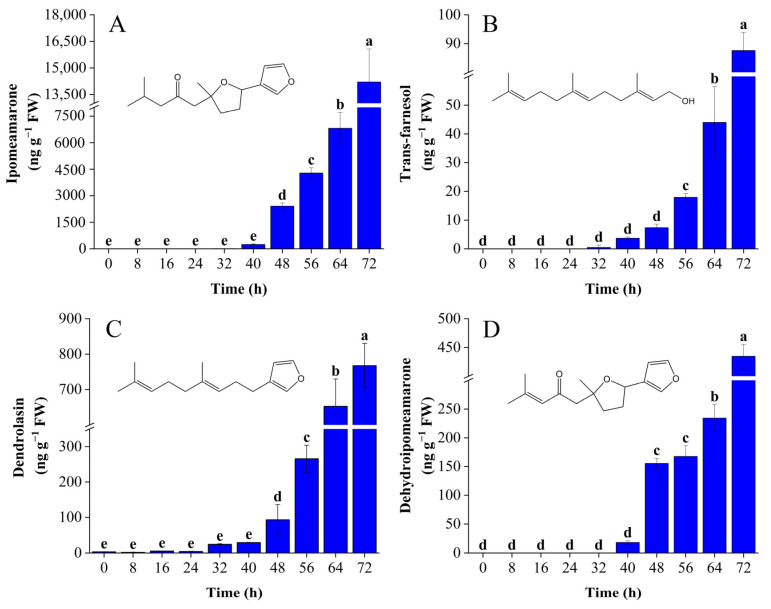
The trends of (**A**) ipomeamarone, (**B**) trans-farnesol, (**C**) dendrolasin, and (**D**) dehydroipomeamarone from 0 to 72 h in *C. fimbriata*-infected sweetpotatoes. Bars represent the standard deviations of three independent measurements. Different lowercase letters represent significant differences according to Duncan’s multiple range test (*p* < 0.05).

Ipomeamarone in ketones is a kind of mycotoxin that belongs to the class of furan terpenoids produced by *C. fimbriata*-infected sweetpotatoes. It can be seen from Figure 5A that ipomeamarone was not detected at 0 h of inoculation. During the early period of infection from 0 to 32 h, the growth of *C. fimbriata* in sweetpotatoes was in the lag phase, resulting in low levels of ipomeamarone, from 0 to 12.93 ng g^−1^ FW. The release of trace amounts may have been due to the stress response generated by mechanical damage at the inoculation holes [46]. From 48 h on, the content of ipomeamarone increased rapidly and reached 14,203.80 ng g^−1^ FW at 72 h, accounting for 81.88%, which was the main component of ketones. Dehydroipomeamarone is a direct precursor for the synthesis of ipomeamarone [47], which is also toxic [48]. Trans-farnesol has been confirmed to be the initial precursor for the synthesis of ipomeamarone, and 6-oxodendrolasin is an intermediate in the synthesis from trans-farnesol to ipomeamarone. Therefore, it can be speculated that dendrolasin may also be one of the intermediates for the synthesis of ipomeamarone according to the structural formula. The trends of trans-farnesol, dendrolasin, and dehydroipomeamarone content from 0 to 72 h are shown in Figure 5B–D. The content of these four substances increased with the prolongation of infection time. Table 2 shows the correlation coefficients between the total colony count of *C. fimbriata* and trans-farnesol, dendrolasin, dehydroipomeamarone, and ipomeamarone. The correlation coefficients were all higher than 0.9, indicating a high connection among the four substances mentioned above in the synthetic pathway. To sum up, even when there are no obvious lesion symptoms on the surface of sweetpotatoes, large amounts of ipomeamarone may have accumulated in the internal infected parts, greatly increasing the risk of toxicity and compromising food safety. Therefore, it is of great significance to explore early discrimination and prediction technology for *C. fimbriata*-infected sweetpotatoes.

### 3.3. E-nose Response Signal for Volatile Compounds in Sweetpotatoes

**Figure 6:**
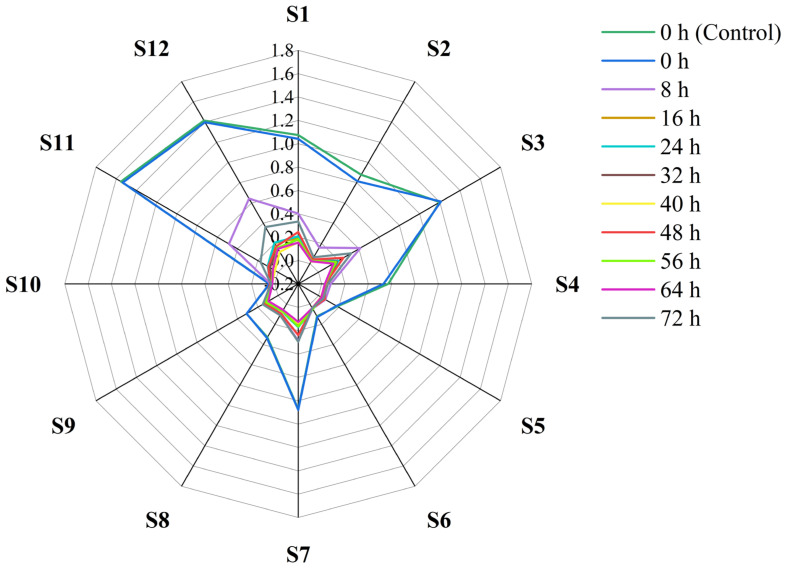
Response of 12 sensors to volatile compounds in sweetpotatoes.

The response signal of each sensor from 0 to 120 s was selected. The response curves of different sensors are shown in Figure S1. After the exponential growth, the sensor had a sufficient response to volatile compounds and reached equilibrium, so the response signal of the sensor reached a steady state in the later stage. During sample measurement, the response signal (V) of each sensor varied with the volatile compounds of sweetpotatoes. It can be seen from Figure 6 that the response signal of sensor S10 was the lowest, followed by S5 and S6. The response signal of sensors S1, S3, S7, S11, and S12 changed the most in different periods, followed by sensors S2, S4, S8, and S9, indicating that these nine sensors were more sensitive to the volatile compounds of sweetpotatoes.

### 3.4. Correlation Analysis between E-Nose and HS-SPME/GC–MS

**Figure 7:**
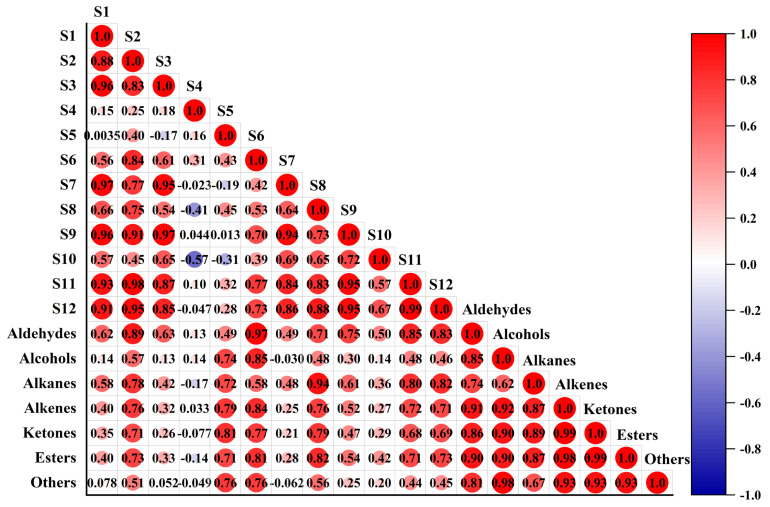
Correlation analysis between sensors and volatile compounds.

HS-SPME/GC–MS mainly focuses on the identification and quantification of a single substance and is highly sensitive and accurate. However, its high operating cost and required personnel input limit its widespread practical application in agriculture [49]. E-nose is economical, fast, non-destructive, and capable of batch analysis. According to the results of HS-SPME/GC–MS, the content of volatile compounds in *C. fimbriata*-infected sweetpotatoes changed greatly from 40 to 72 h due to the proliferation of *C. fimbriata*. Therefore, a correlation analysis between E-nose and HS-SPME/GC–MS from 40 to 72 h was performed, and the results are shown in Figure 7. Table S1 showed the reported sensitive substance of the sensor array in E-nose. Sensors S1 (r = 0.62, *p* < 0.05) and S3 (r = 0.63, *p* < 0.05), which were reported to be sensitive to organic solvents, showed high correlations with aldehydes. S5 (r = 0.72, *p* < 0.05), S8 (r = 0.94, *p* < 0.05), and S9 (r = 0.61, *p* < 0.05), which were reported to be sensitive to alkanes, also showed correlations with alkanes in this study. S11 was reported to be sensitive to volatile organic compounds, and its correlation with aldehydes, alkanes, alkenes, ketones, and esters was 0.85, 0.80, 0.72, 0.68, and 0.71, respectively, in the present study. S6 was reported to be sensitive to alkanes, but we found that it also had a high correlation with aldehydes (r = 0.97, *p* < 0.05) and alcohols (r = 0.85, *p* < 0.05). Although the volatile compounds, including ammonia, amine series, and organic sulfur, were not detected by HS-SPME/GC–MS, sensors S2 and S12 showed responses to these substance in sweetpotatoes. Simultaneously, S2 and S12 had a high correlation with other volatile compounds in sweetpotatoes. The correlations between S2 and aldehydes, aldehydes, alkanes, alkenes, ketones and esters were 0.89, 0.78, 0.76, 0.71, and 0.73, respectively. The correlations between S12 and aldehydes, aldehydes, alkanes, alkenes, ketones, and esters were 0.83, 0.82, 0.71, 0.69, and 0.73, respectively. S4, S7, and S10 had low correlations with various volatile compounds. These three sensors were reported to be sensitive to freon and hydrogen, but these two volatile compounds were not detected by HS-SPME/GC–MS. So, then, the sensors (S4, S7, and S10) showed low correlations with freon and hydrogen in the present work.

Our results indicated that the sensors of E-nose were not only responsive to the specific gases provided by the company standard but were also responsive to other volatile components. The response signals of the sensors showed good correlations with volatile compounds detected by HS-SPME/GC–MS in this study, so that the detection results of E-nose could reflect the changes in volatile components in *C. fimbriata*-infected sweetpotatoes accurately. In addition, compared with a single sensor, E-nose, which is composed of a sensor array, can reflect the changes in sweetpotato volatile components more accurately and comprehensively, which further enhances the reliability of the detection results.

### 3.5. Early Discrimination of C. fimbriata-Infected Sweetpotatoes Combined with Chemometrics

**Figure 8:**
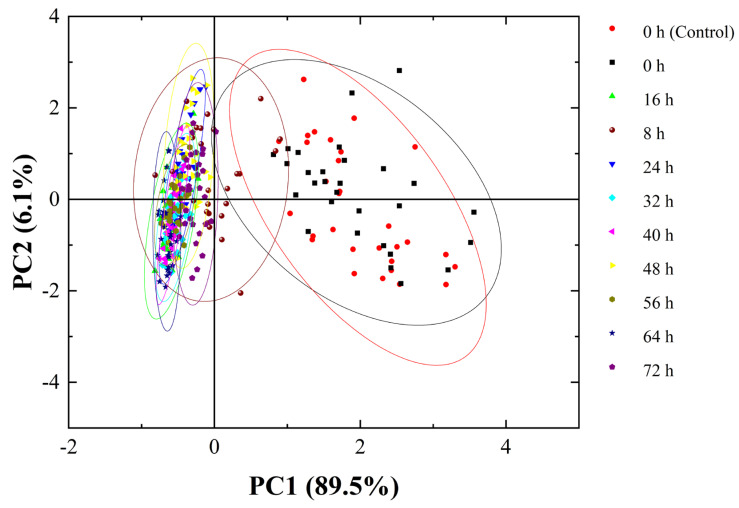
PCA results for the *C. fimbriata*-infected group and the control group from 0 to 72 h.

First, PCA was used to analyse *C. fimbriata*-infected sweetpotatoes from 0 to 72 h. It can be seen from Figure 8 that the first and second PCs explained 89.5% and 6.1% of the variance in the input variables, respectively, with a total of 95.6%. The first two PCs can be considered as comprehensive information for the representation of entire samples [50]. The samples of the *C. fimbriata*-infected group and the control group at 0 h showed a large amount of overlap. This indicated that the volatile compound contents of fresh sweetpotatoes and *C. fimbriata*-infected sweetpotatoes were mostly the same at 0 h, which was consistent with the results of HS-SPME/GC–MS. Based on the results of the two flavor-detection techniques, it can be considered that the *C. fimbriata*-infected group and the control group had the same freshness at 0 h. The fresh samples at 0 h were well distinguished from the infected samples from 8 to 72 h. However, *C. fimbriata*-infected sweetpotatoes from 8 to 72 h were indistinguishable. This was probably because the volatile compound contents did not change significantly in the early period of inoculation. Therefore, four distinguishable time points were screened out: 0 h, 48 h, 64 h, and 72 h. Three models were used to discriminate *C. fimbriata*-infected sweetpotatoes at different infection times: PLS-DA, LDA, and CART.

**Figure 9:**
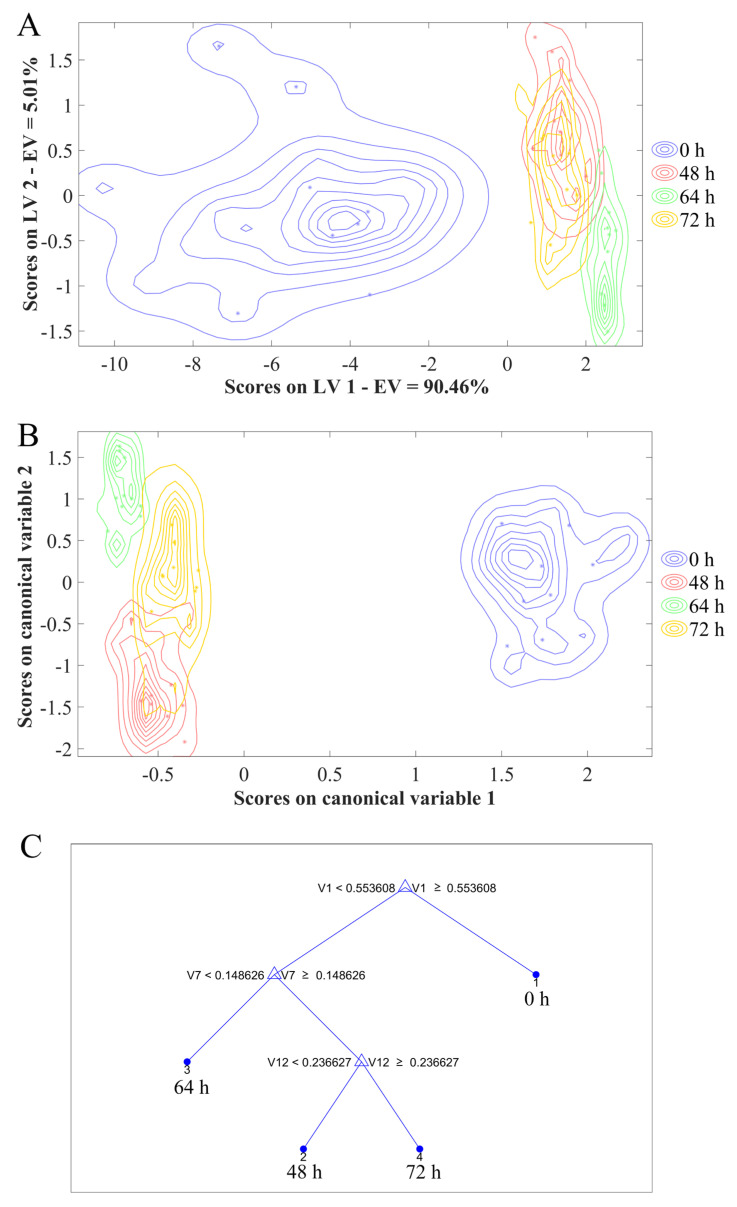
Three discriminant models of *C. fimbriata*-infected sweetpotatoes at 0 h, 48 h, 64 h, and 72 h. (**A**) PLS-DA. (**B**) LDA. (**C**) CART. Solid circles in (**A**) and (**B**) represent the calibration set; the symbol * represents the prediction set.

E-nose data for *C. fimbriata*-infected sweetpotatoes at 0 h, 48 h, 64 h, and 72 h were used for discrimination. The division of the data sets referred to the method of Li et al. [51]; 70% of the data was selected as the calibration set and 30% as the prediction set. The higher the sensitivity, specificity, precision, and accuracy of the models, the better the performance. The results are shown in Figure 9A–C and Table 3. The accuracies of the prediction sets of PLS-DA, LDA, and CART were 100%, 100%, and 97%, respectively. The prediction sets of PLS-DA and LDA had the highest accuracies, both reaching 100%. Among them, the sensitivity, specificity, and precision of LDA at different time points all reached 100%. However, the precision of PLS-DA was lower than that of LDA at 48 h. At the same time, it can be seen from Figure 9A,B that the discrimination result of LDA was better than that of PLS-DA, indicating that LDA can more accurately discriminate *C. fimbriata* infection at different times, followed by PLS-DA. Figure 9C showed that CART could also distinguish sweetpotatoes at different infection times, but its accuracy was lower than LDA and PLS-DA. Simultaneously, the sensitivity of CART was lower than that of PLS-DA at 48 h; the specificity and precision were also lower than those of PLS-DA at 72 h. Among the above three models, LDA had the best discrimination effect, followed by PLS-DA and finally CART. These results showed that E-nose could not only discriminate fresh sweetpotatoes (0 h) from *C. fimbriata*-infected sweetpotatoes but also accurately discriminate infected sweetpotatoes at 48 h, 64 h, and 72 h. At the same time, it also indicated that E-nose can discriminate *C. fimbriata*-infected sweetpotatoes as early as 48 h in the asymptomatic period. At 48 h, the growth of *C. fimbriata* entered into the exponential phase. Slight black lesions could be seen in the internal tissue. Volatile compounds changed significantly (*p* < 0.05), which was consistent with the earliest time point when E-nose could distinguish infected sweetpotatoes from fresh sweetpotatoes.

### 3.6. Prediction of Ipomeamarone Content and Total Colony Count Using E-Nose Combined with Chemometrics

E-nose data for *C. fimbriata*-infected sweetpotatoes at 0 h, 48 h, 64 h, and 72 h were used for prediction. Three models were used to predict: KNN, PLS, and PCR. A model that had higher $R^{2}$ and RPD but lower RMSE (both for calibration and prediction sets) had better performance [52]. When RPD > 3.0, the model had excellent calibrating robustness and high predictive accuracy, which could be applied for actual value prediction [53].

**Figure 10:**
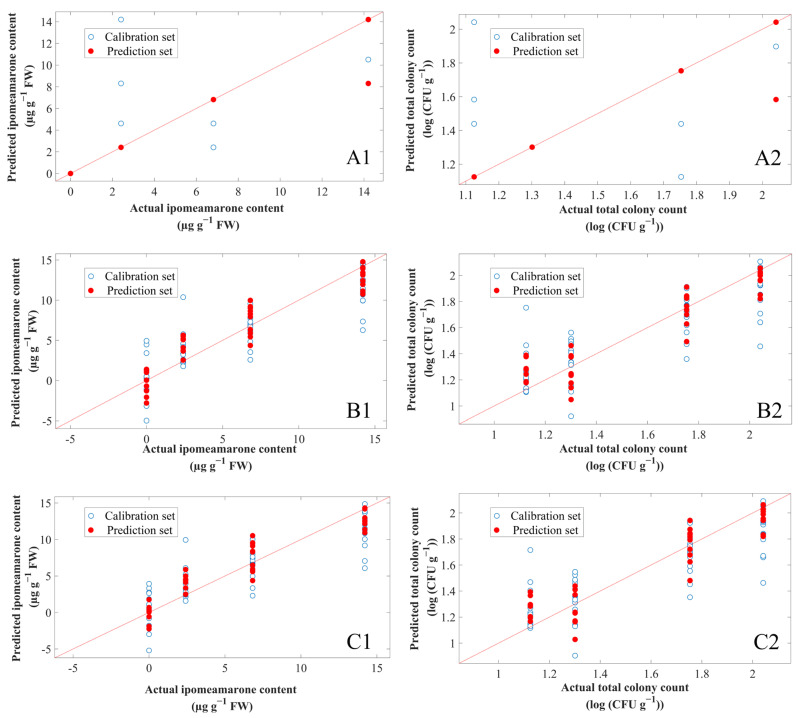
The predicted ipomeamarone content and total colony count based on E-nose data. (**A1**,**A2**) KNN model of (**A1**) ipomeamarone and (**A2**) total colony count. (**B1**,**B2**) PLS model of (**B1**) ipomeamarone and (**B2**) total colony count. (**C1**,**C2**) PCR model of (**C1**) ipomeamarone and (**C2**) total colony count.

The prediction model parameters of ipomeamarone were set as follows. When the number of K neighbours of KNN was 2, the latent variable (LV) of PLS was 7, and the number of PCs of PCR was 10, the cross-validation sets of the three models had the highest $R_{\mathrm{cv}}{}^{2}$ and the lowest RMSE of cross-validation (RMSECV). Therefore, these parameters were used to train the calibration sets and then the prediction sets were used to test models. The prediction results are shown in Figure 10(A1–C1) and Table 4, in which the contents of ipomeamarone are presented as µg g^−1^ FW. The $R_{p}{}^{2}$ of KNN ($R_{p}{}^{2}$ = 0.932) was higher than that of PCR and PLS. The RMSEP of KNN (RMSEP = 1.410 µg g^−1^ FW) was lower than that of PCR and PLS. The RPD of KNN (RPD = 3.852) was also higher than that of PCR and PLS, indicating that KNN had the best highest predictive accuracy, followed by PCR and PLS. The $R_{p}{}^{2}$ of PCR was higher than that of PLS, the RMSEP was lower than that of PLS, and the RPD was higher than that of PLS, indicating that the prediction performance of PCR was better than PLS. In summary, the best model for the prediction of ipomeamarone was KNN, followed by PCR and finally PLS.

The prediction model parameters for the total colony count were set as follows. When the number of K neighbours of KNN was 2, the LV of PLS was 8, and the PCs of PCR was 10, the cross-validation sets of three models had the highest $R_{\mathrm{cv}}{}^{2}$ and the lowest RMSECV. The prediction results are shown in Figure 10(A1–C1) and Table 5, in which the total colony count is presented as log (CFU g^−1^) (the log was base 10). The $R_{p}{}^{2}$ of KNN ($R_{p}{}^{2}$ = 0.909) was higher than that of PLS and PCR. The RMSEP of KNN (RMSEP = 0.110 log (CFU g^−1^)) was lower than that of PLS and PCR. Meanwhile, the RPD of KNN (RPD = 3.306) was higher than that of PLS and PCR, indicating that KNN had the highest predictive accuracy, followed by PLS and PCR. The $R_{p}{}^{2}$ of PLS was higher than that of PCR, the RMSEP was lower than that of PCR, and the RPD was higher than that of PCR, indicating that the prediction performance of PLS was better than that of PCR. To sum up, the best model for the prediction of total colony count was KNN, followed by PLS and finally PCR.

It can be seen from the above that KNN has the best prediction effect on ipomeamarone content and total colony count. In regression by KNN, the input consists of the closest training examples in the data set, and the output is the property value for the object—in other words, the average of the values of K-nearest neighbours [54]. Therefore, distance and K neighbours are the important factor affecting the prediction results of KNN. In this study, Euclidean distance and optimized K neighbours used in the model achieved a good effect in prediction. This also illustrates the feasibility of KNN for predicting sweetpotato toxins and pathogen growth.

## 4. Conclusions

In this study, a self-assembled E-nose was used to evaluate the feasibility of early discrimination and prediction for *C. fimbriata*-infected sweetpotatoes during the asymptomatic period. The results showed that when the sweetpotatoes were infected by *C. fimbriata*, the growth of *C. fimbriata* was in the lag phase before 40 h and entered the exponential phase at 48 h. The total colony count increased rapidly and lesion growth accelerated from 48 h, resulting in significant differences in volatile compound contents at different times, especially in the increase of ipomeamarone. The response signal of the sensors had a good correlation with volatile compounds detected by HS-SPME/GC–MS. The sensor signals combined with PCA, PLS-DA, LDA, and CART were used to discriminate *C. fimbriata*-infected sweetpotatoes. Among them, LDA achieved the best discrimination effect. It could accurately discriminate *C. fimbriata*-infected sweetpotatoes during the asymptomatic period, as early as 48 h after infection, and the accuracy of the prediction set reached 100%. E-nose combined with KNN, PLS, and PCR was used to predict contents of ipomeamarone and total colony counts. In tests of prediction models, the optimal prediction result for ipomeamarone contents was achieved by KNN ($R_{p}{}^{2}$= 0.932, RMSEP = 1.410 µg g^−1^ FW, and RPD = 3.852), and the optimal prediction result for total colony counts was also achieved by KNN ($R_{p}{}^{2}$= 0.909, RMSEP = 0.110 log (CFU g^−1^), and RPD = 3.306).

To the best of our knowledge, there has been no prior study on the volatile compounds associated with sweetpotato disease being detected by HS-SPME/GC–MS and E-nose combined with pathogen growth. This study demonstrated that E-nose could be used as a practical and efficient detection method for *C. fimbriata*-infected sweetpotatoes during the asymptomatic period. In practical applications, ambient gas from different positions in storage warehouses can be collected by gas samplers or pipeline systems and these can be connected to a computer to detect disease in sweetpotatoes online and in real time using E-nose. To sum up, E-nose has great potential application prospects in early disease control and monitoring of agricultural products during storage and transportation.

## Figures and Tables

**Table 1:** Content of different categories of volatile compounds in the *C. fimbriata*-infected group and the control group from 0 to 72 h. (Unit: ng g^−1^ FW equivalent of 2-octanol).

Time (h)	Aldehydes	Alcohols	Alkanes	Alkenes	Ketones	Esters	Others
0 (Control)	496.35 ± 44.56 ^cA^	311.02 ± 61.60 ^dB^	13.34 ± 1.40 ^dD^	134.89 ± 7.25 ^fC^	22.84 ± 3.65 ^eD^	49.98 ± 4.35 ^eD^	3.75 ± 1.21 ^eD^
0	499.00 ± 26.34 ^cA^	296.54 ± 108.77 ^dB^	12.59 ± 1.49 ^dD^	133.39 ± 15.50 ^fC^	23.39 ± 3.18 ^eD^	50.95 ± 14.33 ^eD^	3.18 ± 0.51 ^eD^
8	348.02 ± 24.42 ^deA^	282.95 ± 15.65 ^dB^	19.35 ± 0.80 ^dDE^	140.66 ± 28.37 ^fC^	19.32 ± 5.43 ^eDE^	42.00 ± 8.12 ^eD^	1.68 ± 0.83 ^eE^
16	514.80 ± 120.50 ^cA^	335.73 ± 9.34 ^dB^	82.85 ± 18.66 ^bcdC^	522.37 ± 100.61 ^deA^	28.35 ± 8.50 ^eC^	58.34 ± 6.76 ^deC^	6.50 ± 1.59 ^eC^
24	411.93 ± 79.23 ^cdA^	305.17 ± 11.34 ^dB^	28.90 ± 0.82 ^cdD^	142.50 ± 32.01 ^fC^	22.05 ± 5.81 ^eD^	47.67 ± 7.87 ^eD^	4.07 ± 0.84 ^eD^
32	742.72 ± 50.90 ^bA^	372.05 ± 17.51 ^dB^	39.60 ± 6.09 ^bcdC^	678.17 ± 316.08 ^dA^	100.69 ± 34.94 ^eC^	77.71 ± 6.97 ^cdC^	27.22 ± 6.14 ^eC^
40	241.57 ± 36.99 ^eC^	329.20 ± 33.58 ^dB^	49.58 ± 7.70 ^bcdD^	413.32 ± 45.67 ^eA^	379.07 ± 45.36 ^eAB^	53.48 ± 16.41 ^deD^	32.01 ± 3.87 ^eD^
48	694.50 ± 125.8 ^bBC^	626.53 ± 95.87 ^cC^	100.14 ± 24.43 ^bcD^	903.59 ± 99.88 ^cB^	2932.65 ± 249.65 ^dA^	101.57 ± 15.67 ^cD^	96.02 ± 42.29 ^dD^
56	518.93 ± 43.41 ^cC^	878.19 ± 149.36 ^bB^	110.02 ± 6.67 ^bD^	1112.41 ± 97.31 ^cB^	5131.48 ± 406.33 ^cA^	94.84 ± 4.21 ^cD^	273.18 ± 41.65 ^cCD^
64	721.62 ± 28.65 ^bBC^	1352.64 ± 302.55 ^aB^	56.87 ± 46.74 ^bcdC^	1396.61 ± 109.82 ^bB^	8055.23 ± 1010.49 ^bA^	160.34 ± 6.00 ^bC^	669.30 ± 76.45 ^bBC^
72	963.91 ± 33.46 ^aC^	1473.76 ± 29.78 ^aBC^	376.55 ± 118.15 ^aC^	2304.64 ± 177.67 ^aB^	1,7346.42 ± 1682.93 ^aA^	270.25 ± 34.08 ^aC^	835.11 ± 67.99 ^aC^

Data represent mean values ± standard deviations of three independent measurements. Values with different lowercase letters in the same column are significantly different (*p* < 0.05); values with different capital letters in the same row are significantly different (*p* < 0.05).

**Table 2:** Correlation coefficient between total colony count and trans-farnesol, dendrolasin, dehydroipomeamarone, and ipomeamarone in *C. fimbriata*-infected sweetpotatoes.

	Trans-Farnesol	Dendrolasin	Dehydroipomeamarone	Ipomeamarone
Total colony count	0.978 **	0.937 **	0.940 **	0.968 **
Trans-farnesol	1	0.937 **	0.951 **	0.987 **
Dendrolasin		1	0.929 **	0.933 **
Dehydroipomeamarone			1	0.973 **

The symbol ** indicates that the correlation was significant at a 0.01 confidence level (double test).

**Table 3:** Discrimination results for *C. fimbriata*-infected sweetpotatoes by PLS-DA, LDA, and CART at 0 h, 48 h, 64 h, and 72 h.

Data Set		Calibration				Cross-Validation				Prediction			
Model	Class	Sens(%)	Spec(%)	Prec(%)	Ac(%)	Sens(%)	Spec(%)	Prec(%)	Ac(%)	Sens(%)	Spec(%)	Prec(%)	Ac(%)
PLS-DA	0 h	100	100	100	97	100	100	100	97	100	100	100	100
	48 h	95	100	100		95	100	100		100	100	90	
	64 h	100	98	95		100	98	94		100	100	100	
	72 h	95	98	95		95	98	95		100	100	100	
LDA	0 h	100	100	100	96	100	100	100	94	100	100	100	100
	48 h	95	98	95		95	97	91		100	100	100	
	64 h	100	98	95		100	97	91		100	100	100	
	72 h	90	98	95		81	98	94		100	100	100	
CART	0 h	100	100	100	95	100	100	100	89	100	100	100	97
	48 h	86	98	95		76	95	84		88	100	100	
	64 h	95	100	100		90	97	90		100	100	100	
	72 h	100	95	88		90	94	83		100	96	90	

**Table 4:** Predicted results of ipomeamarone for *C. fimbriata*-infected sweetpotatoes by KNN, PLS, and PCR.

Data Set	Calibration		Cross-Validation		Prediction		RPD
Model	$R_{c}{}^{2}$	RMSEC (µg g^−1^ FW)	$R_{cv}{}^{2}$	RMSECV (µg g^−1^ FW)	$R_{p}{}^{2}$	RMSEP (µg g^−1^ FW)	
KNN	0.836	2.183	0.834	2.200	0.932	1.410	3.852
PLS	0.771	2.581	0.723	2.840	0.875	1.909	2.845
PCR	0.776	2.556	0.724	2.835	0.877	1.891	2.872

**Table 5:** Predicted results for total colony counts for *C. fimbriata*-infected sweetpotatoes by KNN, PLS, and PCR.

Data Set	Calibration		Cross-Validation		Prediction		RPD
Model	$R_{c}{}^{2}$	RMSEC (log (CFU g^−1^))	$R_{cv}{}^{2}$	RMSECV (log (CFU g^−1^))	$R_{p}{}^{2}$	RMSEP (log (CFU g^−1^))	
KNN	0.744	0.184	0.757	0.179	0.909	0.110	3.306
PLS	0.741	0.185	0.644	0.217	0.867	0.133	2.734
PCR	0.735	0.187	0.661	0.212	0.857	0.138	2.635

## Data Availability

The data sets generated for this study are available on request from the corresponding author.

## Supplementary Materials

The following supporting information can be downloaded at: https://www.mdpi.com/article/10.3390/foods11131919/s1, Table S1: The sensor array of E-nose; Table S2: The relative humidity in the air chamber during sample testing using E-nose. Table S3: Contents of different volatile compounds detected and quantified in the *C. fimbriata*-infected group and in the control group by HS-SPME/GC–MS from 0 to 72 h; Figure S1: Response curves from twelve E-nose sensors.

## References

[1] Tsai Y.-J., Lin L.-Y., Yang K.-M., Chiang Y.-C., Chen M.-H., Chiang P.-Y. (2021). Effects of Roasting Sweet Potato (*Ipomoea batatas* L. Lam.): Quality, Volatile Compound Composition, and Sensory Evaluation. Foods.

[2] Sun Y., Pan Z., Yang C., Jia Z., Guo X. (2019). Comparative Assessment of Phenolic Profiles, Cellular Antioxidant and Antiproliferative Activities in Ten Varieties of Sweet Potato (*Ipomoea batatas*) Storage Roots. Molecules.

[3] Alam M.K. (2021). A comprehensive review of sweet potato (*Ipomoea batatas* [L.] Lam): Revisiting the associated health benefits. Trends Food Sci. Technol..

[4] Pang L., Lu G., Cheng J., Lu X., Ma D., Li Q., Li Z., Zheng J., Zhang C., Pan S. (2021). Physiological and biochemical characteristics of sweet potato (*Ipomoea batatas* (L.) Lam) roots treated by a high voltage alternating electric field during cold storage. Postharvest Biol. Technol..

[5] Liu R., Yu Z.-L., Sun Y.-L., Zhou S.-M. (2021). The enzymatic browning reaction inhibition effect of strong acidic electrolyzed water on different parts of sweet potato slices. Food Biosci..

[6] Xu M., Guo J., Li T., Zhang C., Peng X., Xing K., Qin S. (2021). Antibiotic Effects of Volatiles Produced by *Bacillus tequilensis* XK29 against the Black Spot Disease Caused by *Ceratocystis fimbriata* in Postharvest Sweet Potato. J. Agric. Food Chem..

[7] Pang L.-J., Adeel M., Shakoor N., Guo K.-R., Ma D.-F., Ahmad M.A., Lu G.-Q., Zhao M.-H., Li S.-E., Rui Y.-K. (2021). Engineered Nanomaterials Suppress the Soft Rot Disease (*Rhizopus stolonifer*) and Slow Down the Loss of Nutrient in Sweet Potato. Nanomaterials.

[8] Wang C.-J., Wang Y.-Z., Chu Z.-H., Wang P.-S., Liu B.-Y., Li B.-Y., Yu X.-L., Luan B.-H. (2020). Endophytic Bacillus amyloliquefaciens YTB1407 elicits resistance against two fungal pathogens in sweet potato (*Ipomoea batatas* (L.) Lam.). J. Plant Physiol..

[9] Li X., Liu M., Huang T., Yang K., Zhou S., Li Y., Tian J. (2020). Antifungal effect of nerol via transcriptome analysis and cell growth repression in sweet potato spoilage fungi Ceratocystis fimbriata. Postharvest Biol. Technol..

[10] Mohsin S.M., Hasanuzzaman M., Parvin K., Morokuma M., Fujita M. (2021). Effect of tebuconazole and trifloxystrobin on Ceratocystis fimbriata to control black rot of sweet potato: Processes of reactive oxygen species generation and antioxidant defense responses. World J. Microbiol. Biotechnol..

[11] Stahr M., Quesada-Ocampo L.M. (2020). Assessing the Role of Temperature, Inoculum Density, and Wounding on Disease Progression of the Fungal Pathogen Ceratocystis fimbriata Causing Black Rot in Sweetpotato. Plant Dis..

[12] Paul N.C., Nam S.-S., Kachroo A., Kim Y.-H., Yang J.-W. (2018). Characterization and pathogenicity of sweet potato (Ipomoea batatas) black rot caused by Ceratocystis fimbriata in Korea. Eur. J. Plant Pathol..

[13] Tian J., Pan C., Zhang M., Gan Y.Y., Pan S.Y., Liu M., Li Y.X., Zeng X.B. (2018). Induced cell death in *Ceratocystis fimbriata* by pro-apoptotic activity of a natural organic compound, perillaldehyde, through Ca^2+^ overload and accumulation of reactive oxygen species. Plant Pathol..

[14] Zhang Y., Li T., Liu Y., Li X., Zhang C., Feng Z., Peng X., Li Z., Qin S., Xing K. (2019). Volatile Organic Compounds Produced by *Pseudomonas chlororaphis* subsp. *aureofaciens* SPS-41 as Biological Fumigants to Control *Ceratocystis fimbriata* in Postharvest Sweet Potatoes. J. Agric. Food Chem..

[15] Usuki Y., Deguchi T., Iio H. (2014). A New Concise Synthesis of (+)-Ipomeamarone, (−)-Ngaione, and Their Stereoisomers. Chem. Lett..

[16] Boyd M.R., Burka L.T., Harris T.M., Willson B.J. (1974). Lung-toxic furanoterpenoids produced by sweet potatoes (Ipomoea batatas) following microbial infection. Biochim. Biophys. Acta (BBA)-Lipids Lipid Metab..

[17] Pandey G. (2008). Acute toxicity of ipomeamarone, a phytotoxin isolated from the injured sweet potato. Pharmacogn. Mag..

[18] Zhang Y., Li T., Xu M., Guo J., Zhang C., Feng Z., Peng X., Li Z., Xing K., Qin S. (2021). Antifungal effect of volatile organic compounds produced by *Pseudomonas chlororaphis* subsp. *aureofaciens* SPS-41 on oxidative stress and mitochondrial dysfunction of Ceratocystis fimbriata. Pestic. Biochem. Physiol..

[19] Sugimura T., Koguro K., Tai A. (1993). Total syntheses of (+)-ipomeamarone and (−)-ngaione. Tetrahedron Lett..

[20] Wang A., Luca A., Edelenbos M. (2019). Emission of volatile organic compounds from yellow onion (*Allium cepa* L.) bulbs during storage. J. Food Sci. Technol..

[21] Chalupowicz D., Veltman B., Droby S., Eltzov E. (2020). Evaluating the use of biosensors for monitoring of Penicillium digitatum infection in citrus fruit. Sensors Actuators B Chem..

[22] Gong D., Bi Y., Zong Y., Li Y., Sionov E., Prusky D. (2022). Characterization and sources of volatile organic compounds produced by postharvest pathogenic fungi colonized fruit. Postharvest Biol. Technol..

[23] Biancolillo A., Aloia R., Rossi L., D’Archivio A.A. (2021). Organosulfur volatile profiles in Italian red garlic (*Allium Sativum* L.) varieties investigated by HS-SPME/GC-MS and chemometrics. Food Control.

[24] Shen X., Wang Y., Ran L., Liu R., Sun X., Hu L., Xiao Y., Chen F. (2022). Flavor deterioration of liquid endosperm in postharvest tender coconut revealed by LC-MS-based metabolomics, GC-IMS and E-tongue. Postharvest Biol. Technol..

[25] Li J., Xu Y., Du W., Jin L., Ren P., Ren F., Xie J.C. (2022). Comparative analysis of aroma compounds in Chinese traditional dry-rendered fat by HS/GC-IMS, SPME/GC-MS, and SPME/GC-O. J. Food Compos. Anal..

[26] Yu S., Huang X., Wang L., Ren Y., Zhang X., Wang Y. (2022). Characterization of selected Chinese soybean paste based on flavor profiles using HS-SPME-GC/MS, E-nose and E-tongue combined with chemometrics. Food Chem..

[27] Guo Q., Adelina N.M., Hu J., Zhang L., Zhao Y. (2021). Comparative analysis of volatile profiles in four pine-mushrooms using HS-SPME/GC-MS and E-nose. Food Control.

[28] Gu S., Chen W., Wang Z., Wang J., Huo Y. (2020). Rapid detection of Aspergillus spp. infection levels on milled rice by headspace-gas chromatography ion-mobility spectrometry (HS-GC-IMS) and E-nose. LWT.

[29] Ali M.M., Hashim N., Aziz S.A., Lasekan O. (2020). Principles and recent advances in electronic nose for quality inspection of agricultural and food products. Trends Food Sci. Technol..

[30] Rutolo M.F., Clarkson J.P., Covington J.A. (2018). The use of an electronic nose to detect early signs of soft-rot infection in potatoes. Biosyst. Eng..

[31] Huang X., Yu S., Xu H., Aheto J.H., Bonah E., Ma M., Wu M., Zhang X. (2019). Rapid and nondestructive detection of freshness quality of postharvest spinaches based on machine vision and electronic nose. J. Food Saf..

[32] Wen T., Zheng L., Dong S., Gong Z., Sang M., Long X., Luo M., Peng H. (2018). Rapid detection and classification of citrus fruits infestation by Bactrocera dorsalis (Hendel) based on electronic nose. Postharvest Biol. Technol..

[33] Makarichian A., Chayjan R.A., Ahmadi E., Zafari D. (2021). Early detection and classification of fungal infection in garlic (A. sativum) using electronic nose. Comput. Electron. Agric..

[34] Wang L., Hu Q., Pei F., Mugambi M.A., Yang W. (2020). Detection and identification of fungal growth on freeze-dried *Agaricus bisporus* using spectra and olfactory sensors. J. Sci. Food Agric..

[35] Leggieri M.C., Mazzoni M., Fodil S., Moschini M., Bertuzzi T., Prandini A., Battilani P. (2020). An electronic nose supported by an artificial neural network for the rapid detection of aflatoxin B1 and fumonisins in maize. Food Control.

[36] Sanaeifar A., Li X., He Y., Huang Z., Zhan Z. (2021). A data fusion approach on confocal Raman microspectroscopy and electronic nose for quantitative evaluation of pesticide residue in tea. Biosyst. Eng..

[37] Zhang J., Zhang B., Dong J., Tian Y., Lin Y., Fang G., Wang S. (2021). Identification of mouldy rice using an electronic nose combined with SPME-GC/MS. J. Stored Prod. Res..

[38] Lan T., Gao C., Yuan Q., Wang J., Zhang H., Sun X., Lei Y., Ma T. (2021). Analysis of the Aroma Chemical Composition of Commonly Planted Kiwifruit Cultivars in China. Foods.

[39] Zhang D., Ji H., Liu S., Gao J. (2020). Similarity of aroma attributes in hot-air-dried shrimp (Penaeus vannamei) and its different parts using sensory analysis and GC–MS. Food Res. Int..

[40] Pang L., Wang J., Lu X., Yu H. (2008). Discrimination of Storage Age for Wheat by E-Nose. Trans. ASABE.

[41] Marchal P.C., Sanmartin C., Martínez S.S., Ortega J.G., Mencarelli F., García J.G. (2021). Prediction of Fruity Aroma Intensity and Defect Presence in Virgin Olive Oil Using an Electronic Nose. Sensors.

[42] Liu Q., Zhao N., Zhou D., Sun Y., Sun K., Pan L., Tu K. (2018). Discrimination and growth tracking of fungi contamination in peaches using electronic nose. Food Chem..

[43] Aguirre J.S., Koutsoumanis K.P. (2016). Towards lag phase of microbial populations at growth-limiting conditions: The role of the variability in the growth limits of individual cells. Int. J. Food Microbiol..

[44] Wei H., Gu Y. (2020). A Machine Learning Method for the Detection of Brown Core in the Chinese Pear Variety Huangguan Using a MOS-Based E-Nose. Sensors.

[45] Nouri B., Mohtasebi S.S., Rafiee S. (2020). Quality detection of pomegranate fruit infected with fungal disease. Int. J. Food Prop..

[46] Wamalwa L.N., Cheseto X., Ouna E., Kaplan F., Maniania N.K., Machuka J., Torto B., Ghislain M. (2014). Toxic Ipomeamarone Accumulation in Healthy Parts of Sweetpotato (*Ipomoea batatas* L. Lam) Storage Roots upon Infection by *Rhizopus stolonifer*. J. Agric. Food Chem..

[47] Schneider J.A., Lee J., Naya Y., Nakanishi K., Oba K., Uritani I. (1984). The fate of the phytoalexin ipomeamarone: Furanoterpenes and butenolides from Ceratocystis fimbriata-infected sweet potatoes. Phytochemistry.

[48] Inoue H., Oba K., Ando M., Uritani I. (1984). Enzymatic reduction of dehydroipomeamarone to ipomeamarone in sweet potato root tissue infected by Ceratocystis fimbriata. Physiol. Plant Pathol..

[49] Barbosa-Pereira L., Rojo-Poveda O., Ferrocino I., Giordano M., Zeppa G. (2019). Assessment of volatile fingerprint by HS-SPME/GC-qMS and E-nose for the classification of cocoa bean shells using chemometrics. Food Res. Int..

[50] Dittrich I., Gertz M., Maassen-Francke B., Krudewig K.-H., Junge W., Krieter J. (2021). Combining multivariate cumulative sum control charts with principal component analysis and partial least squares model to detect sickness behaviour in dairy cattle. Comput. Electron. Agric..

[51] Li Y., Fei C., Mao C., Ji D., Gong J., Qin Y., Qu L., Zhang W., Bian Z., Su L. (2021). Physicochemical parameters combined flash GC e-nose and artificial neural network for quality and volatile characterization of vinegar with different brewing techniques. Food Chem..

[52] Liu Q., Sun K., Zhao N., Yang J., Zhang Y., Ma C., Pan L., Tu K. (2019). Information fusion of hyperspectral imaging and electronic nose for evaluation of fungal contamination in strawberries during decay. Postharvest Biol. Technol..

[53] Wang S., Hu X.-Z., Liu Y.-Y., Tao N.-P., Lu Y., Wang X.-C., Lam W., Lin L., Xu C.-H. (2021). Direct authentication and composition quantitation of red wines based on Tri-step infrared spectroscopy and multivariate data fusion. Food Chem..

[54] Tedesco D., Moreira B.R.D.A., Júnior M.R.B., Papa J.P., da Silva R.P. (2021). Predicting on multi-target regression for the yield of sweet potato by the market class of its roots upon vegetation indices. Comput. Electron. Agric..

