# Supplementary material for: Early Discrimination and Prediction of C. fimbriata-Infected Sweetpotatoes during the Asymptomatic Period Using Electronic Nose
Source: Foods. 2022 Jun 28;11(13):1919. doi: 10.3390/foods11131919 (PMC9265781; doi:10.3390/foods11131919)
Supplement: Supplementary file 1 [file foods-11-01919-s001.zip › foods-1754591-Supplementary Materials.pdf]

Table S1 The sensor array of the E-nose.

Number	Sensor	Substance Sensitivity
S1	TGS2620	Alcohol, organic solvents
S2	TGS826	Ammonia
S3	TGS822	Organic solvents
S4	TGS832	Freon, R0134a, R-404a, R-407c, R-410
S5	TGS813	Methane, propane, butane
S6	TGS816	Methane, propane
S7	TGS2600	Hydrogen, alcohol
S8	TGS2610	Butane, liquefied petroleum gas
S9	TGS2611	Methane, natural gas
S10	TGS2615	Hydrogen
S11	TGS2602	VOCs, ammonia, hydrogen sulfide
S12	TGS2603	Amine series, organic sulfur compounds
S13	HTU20	Temperature and humidity，-40~105℃，0~100%RH

Table S2 The relative humidity in the air chamber during sample testing using E-nose.

**Time (h)**	**Relative humidity (%)**
0	9.82±0.35
8	11.51±0.99
16	10.71±1.3
24	10.68±0.87
32	10.77±0.87
40	10.64±1.13
48	9.70±0.85
56	11.24±0.94
64	9.97±0.61
72	10.33±2.57

Table S3 Content of different volatile compounds detected and quantified in *C. fimbriata*-infected group and the control group by HS-SPME/GC-MS from 0 to 72 h. (Unit: ng g^{-1} FW equivalent of 2-octanol)

Category	Compound	0 h (Control)	0 h	8 h	16 h	24 h	32 h	40 h	48 h	56 h	64 h	72 h
Aldehydes	(E)-2-Octenal	24.12±1.34^{e}	23.28±2.91^{e}	56.68±7.37^{d}	63.14±19.88^{d}	48.24±1.45^{de}	127.76±33.22^{b}	42.79±3.30^{de}	102.52±15.91^{c}	100.54±11.00^{c}	114.04±8.18^{bc}	152.18±6.23^{a}
	Benzeneacetaldehyde	294.91±35.70^{a}	303.14±14.12^{a}	138.16±9.31^{b}	178.20±90.45^{b}	139.02±26.23^{b}	149.34±10.36^{b}	67.14±6.31^{c}	167.54±21.46^{b}	118.55±13.30^{bc}	161.54±26.86^{b}	175.36±9.38^{b}
	Benzaldehyde	65.47±11.75^{e}	61.13±7.94^{ef}	53.40±13.21^{ef}	146.60±4.17^{bc}	38.33±7.24^{f}	165.81±15.75^{b}	51.73±19.31^{ef}	125.81±23.05^{c}	88.45±7.10^{d}	158.29±6.78^{b}	219.89±4.49^{a}
	Nonanal	61.94±4.93^{de}	62.30±1.71^{de}	47.72±0.82^{e}	55.40±16.21^{de}	94.67±20.83^{b}	173.07±23.94^{a}	23.17±5.17^{f}	88.54±9.84^{bc}	70.83±3.97^{cd}	53.66±5.67^{de}	104.20±5.50^{b}
	(E)-2-Nonenal	14.41±0.94^{f}	12.69±2.76^{f}	13.78±4.22^{f}	20.10±0.13^{ef}	27.53±11.86^{def}	34.83±4.33^{cde}	19.62±7.50^{ef}	47.14±10.46^{bc}	40.80±15.50^{cd}	59.31±9.82^{ab}	70.73±10.18^{a}
	2,4-Nonadienal	0.31±0.54^{c}	0.20±0.35^{c}	1.41±0.73^{c}	4.43±3.36^{bc}	6.72±1.72^{bc}	12.65±1.46^{b}	1.82±0.73^{c}	8.96±15.52^{bc}	8.63±2.32^{bc}	13.47±0.65^{b}	27.99±0.63^{a}
	β-Cyclocitral	4.61±1.14^{c}	4.75±1.30^{c}	4.33±1.54^{c}	9.17±1.32^{bc}	11.3±2.46^{b}	16.39±3.93^{a}	8.56±2.05^{bc}	20.76±1.05^{a}	16.97±2.02^{a}	18.27±2.53^{a}	20.28±5.77^{a}
	(Z)-citral	/	/	7.55±2.05^{c}	2.66±4.61^{c}	6.55±1.84^{c}	3.60±6.24^{c}	2.14±3.71^{c}	70.32±30.30^{a}	28.71±9.17^{b}	67.13±8.38^{a}	72.47±7.15^{a}
	Decanal	21.77±0.99^{c}	21.54±5.60^{c}	19.88±1.95^{c}	28.33±3.52^{bc}	33.76±15.09^{abc}	45.05±2.73^{ab}	16.43±6.21^{c}	48.26±26.39^{a}	28.08±3.72^{bc}	40.72±5.66^{ab}	47.45±7.37^{a}
	Hexadecanal	5.41±1.40^{def}	6.25±1.31^{de}	2.84±0.41^{f}	2.80±0.53^{f}	3.40±0.86^{ef}	6.63±0.13^{d}	3.06±1.13^{f}	9.77±2.07^{c}	6.63±1.31^{d}	32.14±3.66^{b}	39.29±2.19^{a}
	Tetradecanal	3.38±0.61^{d}	3.73±0.75^{d}	2.28±0.77^{d}	3.98±0.01^{d}	2.40±0.18^{d}	7.58±0.40^{c}	5.10±0.23^{c}	4.88±1.89^{d}	10.73±3.64	3.05±0.15^{d}	34.07±2.48

								d		b		a
Alcohols	Linalool	4.06±0.42^{c}	4.44±2.36^{c}	5.49±2.11^{c}	9.61±4.47^{c}	3.49±1.83^{c}	19.41±9.38^{c}	14.21±6.91^{c}	83.41±18.47^{c}	263.73±11.65^{b}	366.97±169.18^{a}	262.64±7.71^{b}
	1-Octanol	16.07±2.24^{c}	15.55±1.15^{c}	15.87±3.70^{c}	21.40±3.89^{c}	19.74±0.30^{c}	54.93±4.34^{a}	19.60±0.74^{c}	39.17±6.55^{b}	35.61±3.42^{b}	39.96±3.31^{b}	48.74±6.49^{a}
	1-Octen-3-ol	36.06±8.75^{bc}	37.1±0.04^{bc}	40.13±13.78^{b}	26.09±0.57^{c}	56.6±1.75^{a}	/	36.86±8.09^{bc}	57.16±10.62^{a}	32.99±6.16^{bc}	29.37±1.47^{bc}	62.43±6.13^{a}
	Citronellol	/	/	/	/	/	/	/	/	70.33±121.81^{c}	371.54±150.66^{b}	435.43±15.44^{a}
	(E)-2-Octen-1-ol	3.85±0.76^{de}	3.89±0.19^{de}	2.13±3.70^{e}	8.70±3.74^{cd}	12.72±2.37^{bc}	17.20±3.16^{ab}	5.50±1.12^{de}	16.21±5.51^{ab}	7.90±0.47^{cd}	15.10±1.91^{ab}	19.82±3.70^{a}
	Phenylethyl Alcohol	96.22±15.47^{bc}	92.96±14.09^{bc}	73.20±13.67^{cd}	89.25±23.98^{bc}	53.47±5.16^{de}	77.95±11.95^{cd}	33.22±14.34^{e}	117.09±14.48^{b}	49.46±15.22^{de}	100.66±4.81^{bc}	195.43±27.85^{a}
	Myrtenol	35.41±3.05^{def}	33.34±3.38^{ef}	31.35±6.71^{f}	53.29±6.11^{bc}	40.21±3.16^{cdef}	44.13±10.12^{cdef}	48.71±8.82^{bcd}	60.00±1.08^{b}	47.32±14.68^{bcd}e	88.24±2.08^{a}	76.44±10.47^{a}
	Nerol	41.66±10.37^{d}	42.83±6.16^{d}	44.61±6.63^{d}	40.32±1.98^{d}	41.35±3.11^{d}	53.84±3.68^{d}	47.00±5.18^{d}	85.30±12.80^{c}	217.48±26.75^{a}	152.31±14.25^{b}	199.43±43.68^{a}
	(Z)-3-Nonen-1-ol	4.74±1.17^{c}	4.92±8.52^{c}	13.00±4.38^{c}	25.36±6.60^{bc}	20.33±2.23^{bc}	34.38±4.41^{bc}	17.85±7.91^{c}	83.48±21.83^{a}	54.37±1.23^{ab}	73.06±14.71^{a}	30.15±52.22^{bc}
	trans-farnesol	/	/	/	/	/	0.48±0.83^{d}	3.69±0.41^{d}	7.37±1.21^{d}	17.91±1.37^{c}	44.01±12.46^{b}	87.58±6.30^{a}
	p-Menth-2-en-7-ol, cis-	72.94±83.70^{a}	61.50±106.53^{a}	57.17±1.34^{a}	61.71±5.82^{a}	57.26±7.18^{a}	69.71±6.21^{a}	102.57±2.50^{a}	77.34±32.21^{a}	81.08±34.12^{a}	71.43±2.02^{a}	55.66±14.66^{a}
Alkanes	1-	/	/	/	/	/	1.25±2.16^{b}	4.14±7.17^{b}	9.12±15.80	/	19.58±33.9	153.72±63.

	Chlorooctadecane								^{b}		2^{b}	54^{a}
	Pentadecane, 3-methyl-	/	/	0.97±0.31^{bc}	1.14±1.97^{bc}	1.36±0.07^{bc}	2.86±1.91^{bc}	1.72±0.45^{bc}	2.03±0.35^{bc}	3.40±1.75^{c}	3.93±1.24^{b}	26.20±3.64^{a}
	Tridecane	/	/	/	2.31±4.00^{c}	/	1.49±2.58^{c}	/	14.79±12.88^{ab}	4.05±0.73^{c}	7.94±1.57^{bc}	19.61±3.77^{a}
	Pentadecane	4.58±1.88^{c}	4.19±1.35^{c}	6.47±0.69^{c}	22.53±6.66^{bc}	8.72±0.52^{c}	10.63±4.33^{c}	11.79±0.87^{c}	39.53±17.5^{b}	42.64±2.09^{b}	/	81.69±47.14^{a}
	Hexadecane	3.84±0.82^{d}	3.08±0.12^{d}	7.58±0.43^{d}	40.71±9.25^{a}	10.88±1.55^{cd}	15.41±0.02^{bcd}	26.44±0.90^{abc}	31.72±5.47^{a}	39.75±5.74^{a}	12.16±1.96^{cd}	30.48±26.40^{ab}
	Heptadecane	4.91±0.51^{e}	5.32±0.32^{e}	4.33±0.57^{ef}	15.52±0.46^{b}	7.95±0.91^{d}	7.42±1.63^{d}	5.49±0.47^{e}	2.95±0.48^{f}	13.63±2.09^{c}	8.35±1.34^{d}	43.51±0.11^{a}
	Tetratetracontane	/	/	/	0.65±1.13^{b}	/	0.54±0.94^{b}	/	/	6.55±2.79^{b}	4.92±8.52^{b}	21.33±10.64^{a}
Alkenes	α-Caryophyllene	8.85±0.19^{c}	8.03±1.93^{c}	4.93±2.66^{c}	10.08±3.13^{c}	5.96±2.58^{c}	14.51±9.86^{c}	10.95±2.35^{c}	27.30±5.44^{c}	56.19±33.83^{b}	13.52±23.41^{c}	92.53±1.27^{a}
	Alloaromadendrene	2.73±0.13^{c}	2.49±4.31^{c}	2.69±4.66^{c}	34.42±20.31^{bc}	/	38.58±66.82^{bc}	15.87±6.58^{bc}	11.45±19.84^{bc}	57.42±32.22^{ab}	53.27±10.84^{abc}	97.01±24.76^{a}
	(-)-Aristolene	4.74±1.15^{c}	4.58±1.51^{c}	3.60±1.32^{c}	5.41±1.98^{c}	3.66±1.76^{c}	8.44±5.91^{c}	7.38±0.84^{c}	6.64±11.50^{c}	27.84±15.35^{b}	19.98±3.69^{b}	56.63±4.29^{a}
	Caryophyllene	4.00±1.89^{c}	3.75±0.95^{c}	4.00±0.31^{c}	11.47±0.61^{bc}	4.70±8.14^{c}	7.28±2.46^{c}	6.76±0.03^{c}	20.86±0.81^{b}	6.27±10.85^{c}	1.27±2.20^{c}	77.14±16.36^{a}
	β-Sesquiphellandrene	/	/	6.72±11.64^{c}	/	11.81±1.59^{c}	110.56±83.98^{b}	116.59±21.51^{b}	193.52±38.16^{a}	210.23±8.03^{a}	148.14±11.68^{ab}	38.32±66.38^{c}
	α-Gurjunene	64.69±6.57	65.25±6.91	50.15±7.82	238.92±93.	14.09±5.10	276.10±13	115.18±15.	158.30±35.	205.18±16.	191.15±39.	417.25±35.

	^{ef}	^{ef}	^{f}	11^{bc}	^{f}	9.58^{b}	06^{def}	30^{cde}	.00^{bcd}	27^{bcd}	55^{a}
Cedrene	/	/	/	7.22±12.51^{c}	4.30±7.45^{c}	5.18±8.97^{c}	/	/	/	42.81±1.84^{b}	148.13±23.82^{a}
(+)-Valencene	/	/	/	/	/	/	/	2.16±3.75^{c}	6.95±3.18^{c}	18.77±11.28^{b}	28.32±2.25^{a}
α-Selinene	1.19±2.06^{d}	1.00±1.73^{d}	3.85±1.44^{d}	26.62±0.47^{d}	6.93±4.11^{d}	9.79±0.70^{d}	22.64±1.60^{d}	165.43±21.23^{c}	141.89±5.34^{c}	237.27±76.50^{b}	447.81±22.16^{a}
γ-Muurolene	4.75±0.46^{cd}	4.09±0.33^{cd}	4.45±0.27^{cd}	3.23±5.60^{d}	3.80±2.29^{cd}	5.61±2.13^{cd}	2.15±3.72^{d}	3.63±0.25^{d}	9.34±0.39^{c}	45.92±3.32^{b}	75.73±5.48^{a}
α-Guajene	5.36±1.83^{cd}	5.51±1.86^{cd}	3.36±1.91^{cd}	5.98±1.33^{cd}	2.80±2.64^{d}	10.12±7.71^{c}	6.01±2.49^{cd}	25.89±2.68^{b}	9.84±3.11^{cd}	22.29±3.95^{b}	54.71±5.81^{a}
α-Bergamotene	/	/	/	/	0.78±1.35^{e}	0.91±1.58^{e}	3.62±1.18^{e}	65.35±3.88^{d}	110.53±2.46^{c}	207.04±12.03^{a}	171.63±26.87^{b}
β-Cubebene	2.51±0.36^{f}	2.40±0.98^{f}	2.27±0.96^{f}	7.13±1.88^{de}	4.10±1.69^{ef}	5.39±0.89^{ef}	4.81±2.58^{ef}	31.14±0.16^{b}	9.69±1.91^{cd}	13.63±3.80^{c}	36.54±5.35^{a}
β-Elemene	5.76±0.69^{e}	5.36±0.62^{e}	6.27±1.07^{e}	17.88±3.31^{d}	6.32±2.15^{e}	12.27±1.16^{de}	8.44±0.30^{e}	32.29±8.20^{c}	38.33±5.41^{c}	83.93±0.56^{b}	125.35±6.39^{a}
α-Copaene	10.24±0.45^{d}	10.91±0.64^{d}	9.90±0.51^{d}	31.57±7.28^{c}	7.53±0.03^{d}	12.46±1.25^{d}	11.93±1.33^{d}	23.97±7.13^{c}	25.80±2.97^{c}	44.77±1.87^{b}	88.50±18.76^{a}
α-Cubebene	/	/	0.86±1.49^{fg}	4.44±0.57^{de}	1.07±1.85^{fg}	7.81±3.61^{bc}	2.81±0.93^{ef}	5.90±0.29^{cd}	6.18±0.94^{cd}	9.66±0.54^{b}	15.51±1.82^{a}
Cyclosativene	5.52±1.93^{e}	7.60±2.17^{e}	6.45±1.11^{e}	37.70±3.30^{d}	25.01±8.32^{de}	29.33±8.18^{de}	35.84±7.66^{d}	45.43±10.42^{cd}	66.14±15.14^{c}	117.97±6.67^{b}	255.96±40.02^{a}
1,3-Hexadiene, 3-ethyl-2-methyl-	14.55±2.79^{f}	12.41±2.00^{f}	29.42±9.08^{e}	49.80±9.84^{cd}	39.31±0.74^{de}	94.45±10.01^{a}	30.86±0.24^{e}	59.19±9.11^{c}	50.77±7.68^{cd}	74.88±7.35^{b}	59.85±6.34^{c}
(-)-α-Panasinsen	/	/	1.74±3.02^{c}	30.50±16.3	0.31±0.53^{c}	29.38±50.8	11.49±10.6	25.12±1.18	73.83±38.1	50.36±16.1	17.73±1.80

					1^{bc}		9^{bc}	8^{bc}	^{bc}	8^{a}	0^{ab}	^{bc}
Ketones	(E)-β-Ionone	22.84±3.65^{bc}	23.39±3.18^{bc}	16.47±4.89^{bc}	19.03±6.14^{bc}	17.53±5.39^{bc}	87.76±35.5^{a}	16.49±4.56^{bc}	46.21±21.54^{b}	10.11±17.51^{c}	41.02±3.36^{bc}	19.59±33.94^{bc}
	Artemisia ketone	/	/	/	/	/	/	/	8.54±14.79^{b}	8.87±15.36^{b}	12.74±3.68^{b}	54.47±29.53^{a}
	Ipomeamarone	/	/	2.85±1.72^{e}	9.33±2.90^{e}	4.52±0.58^{e}	12.93±3.16^{e}	244.52±33.80^{e}	2408.82±178.61^{d}	4283.17±296.03^{c}	6817.61±892.87^{b}	14203.80±1865.18^{a}
	2-Hexanone, 1,1,1-trifluoro-	/	/	/	/	/	/	/	6.72±1.19^{cd}	19.33±4.07^{bc}	23.58±11.40^{b}	196.03±25.67^{a}
	dehydroipomeamarone	/	/	/	/	/	/	18.29±3.03^{d}	155.34±9.02^{c}	167.66±18.52^{c}	234.27±23.56^{b}	434.68±20.80^{a}
	1-Acetyl-3-hydroxyadamantane	/	/	/	/	/	/	98.18±7.10^{e}	286.37±156.33^{d}	634.89±98.68^{c}	899.77±140.03^{b}	2350.65±265.49^{a}
	(6E)-9-(3-Furyl)-2,6-dimethylnona-2,6-diene-4-one	/	/	/	/	/	/	1.58±1.41^{b}	20.65±22.94^{b}	7.47±6.65^{b}	26.24±45.45^{b}	87.2±29.07^{a}
Esters	Benzeneacetic acid, ethyl ester	19.57±3.11^{ab}	19.40±8.34^{ab}	10.46±3.64^{c}	15.64±3.82^{bc}	10.02±1.15^{c}	12.88±3.31^{bc}	13.20±0.74^{bc}	17.31±1.95^{b}	12.99±1.64^{bc}	15.89±1.94^{bc}	25.21±2.85^{a}
	Ethyl palmitate	12.59±2.63^{d}	12.83±3.63^{d}	12.29±5.05^{d}	23.23±4.92^{cd}	23.53±8.30^{cd}	36.82±6.07^{bc}	20.64±9.37^{d}	46.71±11.34^{b}	44.80±0.30^{b}	78.78±7.26^{a}	89.95±16.06^{a}
	Methyl palmitate	0.42±0.73^{f}	0.52±0.91^{f}	4.27±0.43^{e}	/	2.67±0.32^{ef}	7.09±0.54^{cd}	5.04±1.87^{de}	9.57±1.03^{c}	8.16±0.02^{c}	16.91±2.50^{b}	31.89±3.73^{a}
	Dibutyl phthalate	6.75±1.29^{c}	7.93±3.21^{b}	3.03±1.53^{c}	7.02±1.60^{c}	3.26±1.40^{c}	5.18±2.77^{c}	4.86±2.61^{c}	8.03±3.79^{b}	8.80±0.23^{b}	13.92±1.40	16.25±8.51

			^{c}						^{c}	^{c}	^{ab}	^{a}
	Diisobutyl phthalate	4.82±1.42^{d}	4.29±0.97^{d}	3.32±0.35^{d}	4.57±1.18^{d}	2.88±0.28^{d}	2.81±0.26^{d}	3.66±1.77^{d}	5.26±0.32^{d}	12.00±1.32^{c}	17.60±1.79^{b}	57.20±4.68^{a}
	Isopropyl Myristate	1.91±1.27^{bc}	1.65±0.33^{c}	5.30±1.06^{bc}	1.45±0.20^{c}	1.59±0.22^{c}	2.03±0.26^{bc}	2.05±0.88^{bc}	5.72±1.11^{bc}	3.41±0.71^{bc}	7.60±1.75^{b}	22.13±9.74^{a}
	Dihydroactinolide	3.92±0.70^{b}	4.32±0.85^{b}	3.34±1.33^{b}	6.42±1.44^{b}	3.73±0.15^{b}	10.9±1.57^{b}	4.02±1.14^{b}	8.98±1.82^{b}	4.68±1.21^{b}	9.64±0.71^{b}	27.61±19.96^{a}
Others	Dendrolasin	3.75±1.21e	3.18±0.50^{e}	1.68±0.83^{e}	5.45±0.26^{e}	4.07±0.84^{e}	24.27±1.14^{e}	29.41±0.75^{e}	93.15±43.08^{d}	265.75±38.51^{c}	652.54±77.22^{b}	767.37±62.87^{a}
	Sesquirosefuran	/	/	/	/	/	/	/	/	/	9.63±1.31^{b}	34.26±0.45^{a}
	Cada-1,4-diene	/	/	/	1.05±1.81^{b}	/	2.96±5.12^{b}	2.60±4.50^{b}	2.87±0.79^{b}	7.43±3.13^{b}	7.13±1.35^{b}	33.48±10.58^{a}

Data represent mean values ± standard deviations of three independent measurements. Values with different lowercase letters in the same row are significantly different ($p < 0.05$).

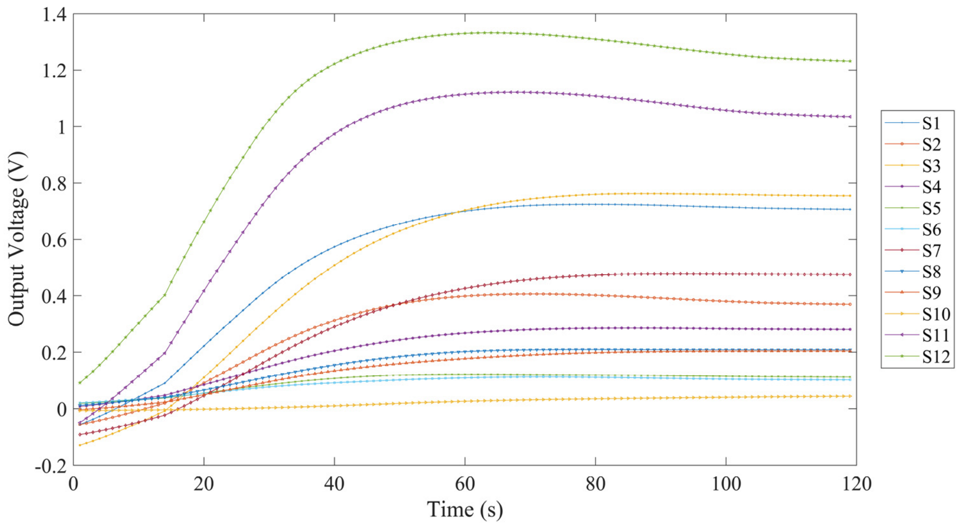

Figure S1. Response curves from twelve sensors of E-nose. The abscissa represents time (s), the ordinate represents the response signal (V), and each curve represents the response change of a sensor within 120 s.
